# Yogurts Supplemented with Juices from Grapes and Berries

**DOI:** 10.3390/foods9091158

**Published:** 2020-08-21

**Authors:** Dimitra Dimitrellou, Nikoletta Solomakou, Evangelos Kokkinomagoulos, Panagiotis Kandylis

**Affiliations:** Department of Food Science and Technology, School of Agriculture, Aristotle University of Thessaloniki, P.O. Box 235, 54124 Thessaloniki, Greece; dimitrellou@gmail.com (D.D.); nsolomak@gmail.com (N.S.); ekokkinom@gmail.com (E.K.)

**Keywords:** total phenolic content, antioxidant activity, DPPH^●^, syneresis, color, aronia, blueberry, grape, yogurt

## Abstract

Nowadays, there is growing interest for the development of enriched dairy products with phenolic compounds derived from edible sources, mainly due to their safety and potential health benefits. Following that trend, in the present study, fruit juices (blueberry, aronia, and grape) were supplemented into yogurt as functional ingredients. The main physicochemical characteristics (pH, reducing sugars, acidity, color, and syneresis), total phenolic content, antioxidant activity, and viability of yogurt starters were monitored during production and storage. The use of juices had no significant effect on milk acidification rate and on the main physicochemical characteristics of yogurts, while resulted in increased red color. Total phenolic content increased from 30 to 33% (grape and aronia) and up to 49% (blueberry), while similar results were observed in antioxidant activity. Similar values of syneresis were presented in all yogurts, probably due to exopolysaccharide producing starter culture. *Streptococcus thermophilus* retained high viable counts during storage especially in yogurts with fruit juices (>10^8^ cells g^−1^) revealing a possible prebiotic effect of juices. The results obtained from this study show that fruit juices (aronia, blueberry, and grape) have potential to be used in yogurt production in order to optimize the benefits of probiotic products with high phenolic compound intake.

## 1. Introduction

In recent years, there is growing consumer interest for health-enhancing foods like low fat products, products with added fiber, and functional foods [1]. At the top of consumers’ preferences, among the functional foods, are dairy products [1,2], which are considered as the ideal host of functional ingredients and, therefore, their functional properties have been extensively investigated [3,4,5]. However, it should be mentioned that, nowadays, there is a growing trend to avoid dairy products due to increase in allergenicity towards cow’s milk, lactose intolerance, vegetarianism, and the prevalence of hypercholesterolemia and other health problems [6]. Lactose intolerance is considered the most usual health problem associated with the consumption of dairy products [7] but also β-lactoglobulin, caseins, and other cow milk proteins are regarded as major allergens [8,9]. In addition, the consumption of high fat milk has been related to hormone-dependent cancers [10]. Nevertheless, yogurt, with a more than 100 year history, is still the leader of dairy products with relatively high consumption and numerous health benefits [11]. Therefore, the acceptance of functional dairy products by consumers remains very high and especially older people and females of all ages have expressed their willingness to incorporate them in their diet [1].

Dairy products like yogurts, fermented milks, and beverages have been characterized as suitable vehicles for the delivery of probiotic bacteria in humans and they have been part of the human diet for many years [12]. In addition, fruit berries and grapes contain the best dietary sources of bioactive compounds [13] that have been associated with the prevention of several chronic diseases [14,15,16,17,18]. Therefore, the supplementation of fermented dairy products with phenolic-rich products like berry and grape juices seems an ideal method to optimize the benefits of probiotic products with high phenolic compound intake. However, yogurts have unique characteristics which make them accepted by the consumers and, therefore, there is a need to clarify if the addition of grape and berry juices may alter them either positively or negatively. The addition of such products is expected to modify some specific aspects of fermented dairy products, such as acidification rate, fermentation time, growth of starter bacteria, and main physicochemical characteristics.

Syneresis or whey separation is one of the most common defects in set type yogurts. This defect may become more serious in the case of low fat yogurts or yogurts produced with the addition of fruit juices [19,20]. Since the use of additives (gelatin, starch, or gums) is not favored by the consumers, the exopolysaccharides (EPSs) produced by lactic acid bacteria may be the solution. Exopolysaccharides are long-chain polysaccharides consisting of branched, repeating units of sugars or sugar derivatives. *Streptococcus thermophilus* is widely used in dairy industry and several strains are able to synthesize EPSs. *Streptococcus thermophilus* EPSs are built from repeating subunits of 3–8 different monosaccharides and are predominantly composed of D-galactose, D-glucose, L-rhamnose, and N-acetyl-galactosamine (GalNAc) in different ratios [21]. Several studies with EPS-producing *S. thermophilus* strains revealed that yogurts presented higher water holding capacity, increased proteolysis, reduced firmness and, in general, improved viscosity, texture, and mouthfeel [22,23,24]. In addition, EPSs from *S. thermophilus* have antibacterial and antioxidant activities and several other potential health benefits [21].

Although the utilization of blueberries juice [25], grape juice [19,26,27], and black chokeberry *Aronia melanocarpa* juice [28] in yogurts and other fermented milks has already been the target of investigations, there are no studies providing a complete evaluation and comparison of the effects of these juices on main characteristics (starter culture viability, physicochemical characteristics, phenolic content, and antioxidant activity) of yogurts and especially during processing and storage. Therefore, the aim of the present study was to incorporate grape and berry juices in yogurts, produced by an EPS-producing starter culture, and to evaluate the physicochemical characteristics, color, functional characteristics, and yogurt starter survival during processing and storage for up to 4 weeks. 

## 2. Materials and Methods 

### 2.1. Milk, Juices and Starter Culture

Commercial pasteurized and homogenized cow’s milk (Koukakis Farm, Greece) with 39 g L^−1^ fat, 47 g L^−1^ sugar and 34 g L^−1^ protein; commercial pasteurized natural juices (no preservatives and no added sugars) from aronia (Aronia Konstantinidis, Greece; 126 g L^−1^ sugar); and blueberries (Zoi, Greece; 100 g L^−1^ sugar) were employed in the present study. In addition, concentrated red grape juice was also employed after dilution with water to 22 °Brix (220 g L^−1^ sugar). Commercial skim milk powder (Regilait, France) with the following content (in 100 g) was also used: 0.8 g fat, 52 g sugar, and 36 g protein. Lyofast Y 436 A (Sacco Systems, Cadorago, Italy) was used as starter culture for yogurt production. This starter consists of exopolysaccharide (EPS)-producing strains of *Streptococcus thermophilus* and a low content of *Lactobacillus delbrueckii* ssp. *bulgaricus*.

### 2.2. Yogurt Production

**Figure 1:**
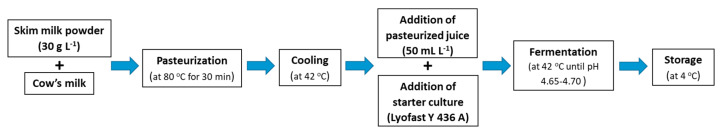
Protocol for the manufacture of yogurts supplemented with juices from grapes and berries.

Yogurt was produced following the procedure described in Figure 1. Briefly, skim milk powder was added in milk (30 g L^−1^) and the mixture was pasteurized at 80 °C for 30 min. After cooling to 42 °C, aronia juice, blueberry juice, and grape juice were added separately to a final concentration of 50 mL L^−1^ in order to produce aronia (AY), blueberry (BY), and grape yogurt (GY), respectively. At this point, the starter culture (Lyofast Y 436 A, Sacco Systems) was added and the inoculated mixture was incubated at 42 °C until the pH reached the value of 4.65–4.70. For comparison, a control yogurt (CY) was produced replacing the pasteurized juice with cow’s milk.

### 2.3. Analyses

#### 2.3.1. Total Acidity and pH

Titratable acidity was determined as % *w*/*w* lactic acid by titrating with 0.1 N NaOH and using phenolphthalein as an indicator. The pH was measured using a portable, electronic pH meter (SensoDirect pH 110, AQUALYTIC, Dortmund, Germany).

#### 2.3.2. Water Holding Capacity and Syneresis

A sample of about 10 g of yogurt (Y) was centrifuged for 10 min at 5000× *g* and at 20 °C. The whey expelled (WE) was removed and weighed. The water holding capacity (WHC) was defined as $WHC\;\left(\%\right)=100\;\frac{\left(Y-WE\right)}{Y}$, where *WE* = whey expelled in g and *Y* = initial yogurt sample in g. Syneresis was determined using 50 mL of unstirred yogurt spread evenly on a Whatman No.1 filter paper (Whatman Ltd., Maidstone, UK) in a funnel at 4 °C. After 5 h of drainage, the volume of whey collected in a beaker, measured, multiplied by 2, and expressed as syneresis (%) [29].

#### 2.3.3. Color

Color characteristics were determined by a colorimeter (CR-400 Chroma Meter, Konica Minolta Inc., Osaka, Japan). CIE L*a*b* color coordinate system used for the analysis. The L* value is a measure of lightness, ranging from blackness (0) to whiteness (100); the a* value ranges from greenness (−60) to redness (+60); and the b* value ranges from blueness (−60) to yellowness (+60). The chroma (C*, brightness) and hue angle were calculated using the following equations: $C^{*}=\;\sqrt{\left(a^{{*}^{2}}+\;b^{{*}^{2}}\right)}$ and $hue=arctan\left(\frac{b^{*}}{a^{*}}\right)$, respectively. The values of hue angle provide details of different colors, as follows: 0° = red-purple, 90° = yellow, 180° = bluish-green, and 270° = blue [30].

#### 2.3.4. Preparation of Yogurt Water Extract

Yogurt samples were diluted and homogenized with distilled water in the ratio of 1:0.25. The pH of the yogurt solution was then adjusted to pH 4.0 with 1.0 N HCl to reduce the solubility of casein in milk. The yogurt was incubated at 45 °C water bath for 10 min. The yogurt was centrifuged at 5000 rpm and 4 °C for 10 min to remove precipitated milk proteins. The pellet was discarded and 0.5 N NaOH was then added to neutralize the supernatant back to pH 7.0 followed by a second centrifugation at 5000 rpm for 10 min at 4 °C for further precipitation of proteins and salts [31]. The supernatant from the second centrifugation was used in the analysis of the following assays.

#### 2.3.5. Free Radical-Scavenging Activity

Free radical-scavenging activity was determined using the free radical DPPH^●^ (2,2 diphenyl-1-picrylhydrazyl) method [32], with some modifications. Specifically, in a 5 mL Eppendorf tube (Eppendorf, Hamburg, Germany), yogurt water extract was diluted with distilled water to a total volume of 1.25 mL, and 3.75 mL of DPPH^●^ solution was added, vortexed, and allowed to stand at room temperature in darkness for 30 min. The absorbance of samples and blank (distilled water instead of sample) were recorded at 515 nm in a UV/Vis spectrophotometer (UV-1800, Shimadzu, Kyoto, Japan) and quantified using Trolox as a standard. 

#### 2.3.6. Total Phenolic Content

Total phenolic content (TPC) was determined by the Folin–Ciocalteu method [32], with some modifications. Specifically, in a 5 mL Eppendorf tube (Eppendorf), water extract was diluted with distilled water to a total volume of 1 mL, 3 mL of distilled water, and 250 μL of Folin–Ciocalteu reagent was added and vortexed. After 1 min, 750 μL of 20% *w*/*w* sodium carbonate was added, vortexed, and allowed to stand at room temperature in darkness, for 120 min. The absorbance was recorded at 750 nm in a UV/Vis spectrophotometer (UV-1800, Shimadzu) and quantified using gallic acid as a standard.

#### 2.3.7. Reducing Sugars

Reducing sugars (RS) were determined using the DNS (3,5-dinitrosalicylic acid) method [33]. Specifically, in a glass test tube, yogurt water extract was diluted with distilled water to a total volume of 500 μL, and 500 μL of DNS solution (1% *w*/*v* 3,5-dinitrosalicylic acid, 30% *w*/*v* potassium sodium tartrate, 1.6% *w*/*v* NaOH) was added, followed by vortexing, and then placed in a water bath at 100 °C for 5 min. The tubes were then cooled to room temperature and 5 mL of distilled water was added. The tubes were vortexed, and absorbance was recorded at 540 nm in a UV/Vis spectrophotometer (UV-1800, Shimadzu) and quantified using D-lactose as a standard.

### 2.4. Microbiological Analysis

Ten grams of each yogurt sample were diluted with 90 mL of sterile Ringer solution and mixed uniformly with a vortex mixer. Subsequent serial dilutions were made, and viable yogurt bacteria were enumerated using the pour plate technique. The counts of *S. thermophilus* were enumerated on M-17 agar (supplemented with 10% *w*/*v* lactose solution) after incubating the plates aerobically at 37 °C for 48 h, while those of *L. delbrueckii* subsp. *bulgaricus* on MRS agar were adjusted to pH 5.4 along with anaerobic incubation at 37 °C for 72 h [34]. Plates containing 30–300 colonies were enumerated and recorded as CFU g^−1^ of sample.

### 2.5. Statistical Analysis

All experiments were carried out in duplicate and duplicate or triplicate samples were collected for each analysis. Significance was established at *p* < 0.05. The results were analyzed for statistical significance with ANOVA, and Tukey’s honest significant difference (HSD) test was used to determine significant differences among results; coefficients, ANOVA tables, and significance (*p* < 0.05) were computed using Statistica Version 5.0 (StatSoft Inc., Tulsa, OK, USA).

## 3. Results and Discussion

### 3.1. Acidification Trend

**Figure 2:**
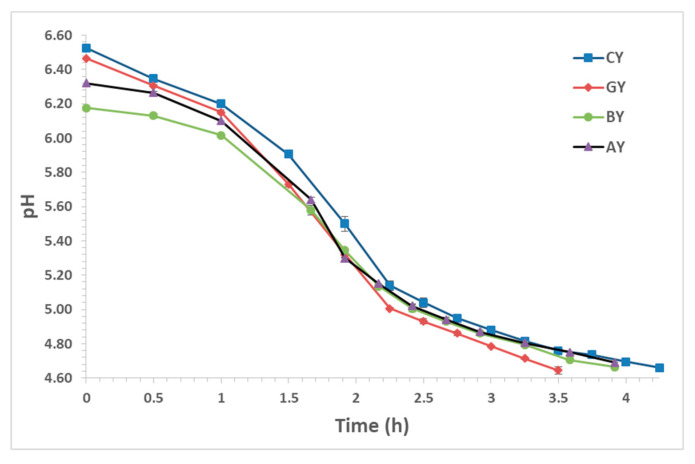
Acidification kinetics during yogurt production (CY = control yogurt; GY = yogurt with grape juice; BY = yogurt with blueberry juice; AY = yogurt with aronia juice).

The acidification trend in yogurt fermentation is presented in Figure 2. The addition of fruit juices affected significantly (*p* < 0.05) the initial pH of milk. More specifically, grape juice resulted in a pH of 6.46, while aronia and blueberry in 6.32 and 6.18, respectively. These values are attributed to the low pH value of the juices used. The maximum rate of pH decline, in all cases, occurred between 1 and 2.5 h after milk inoculation, which is faster compared to previous studies [35] and may be attributed to different starter culture. The use of different juices had no significant effect on the milk acidification rate and the small differences observed were not important from a technological point of view. However, there was a significant reduction in fermentation time up to 45 min compared to the CY. The total acidification time was 4.25, 3.92, 3.92, and 3.5 h for CY, BY, AY, and GY, respectively, showing a reduction with the use of juices. These results are in accordance with previous studies, where higher acidification rate and lower time to coagulation were reported by using aronia and blueberry juices in yogurt production [25,28]. The use of grape juice resulted in the lowest fermentation time among the fruit juices of the present study, and this may be attributed to the higher sugar content (especially glucose and fructose) of the grape juice. However, in previous studies with concentrated grape juice in yogurts, a slower acidification was reported [19,26]. This effect may be attributed to the increased sugar content (causing osmotic stress to the starter culture) and the acidity of grape juice after condensation. 

### 3.2. Physicochemical Characteristics and Antioxidant Activity

As expected, a post-acidification effect was observed in yogurts, resulting in lower pH values during storage (Table 1). In all cases, the effect of storage time was significant (*p* < 0.001), however, the use of fruit juices did not affect the pH of yogurts during storage, especially after 14 days. All yogurt formulations resulted in similar pH values at the end of storage (28 days), ranging 4.36–4.39. These results are important since they indicate that the addition of fruit juices did not affect the fermentation process of yogurt starters. Similar results with those of pH were also reported in the case of acidity in the present study. More specifically, no significant differences in acidity were observed between the different yogurt formulation (CY, GY, AY, and BY) after the 14th day and up to the end of storage. The fermentation process was also monitored by measuring the residual sugar in yogurts. The addition of fruit juices significantly increased the initial sugar content of milk. More specifically, control milk had a sugar content of 5.95% *w*/*v* and the addition of blueberry and aronia juice increased this value to 6.45 and 6.58% *w*/*v*, respectively. A more significant increase was observed in the case of grape juice, resulting in 7.05% *w*/*v*. These variations may be attributed to the different sugar content of each juice. Grape juice contained almost double sugars (22 °Brix) compared to aronia (12.6 °Brix) and blueberry juice (10 °Brix). Storage significantly (*p* < 0.001) affected the sugar content of all yogurts, and despite the previously mentioned differences in initial sugar content, the final sugars in CY, AY, and BY were similar (*p* < 0.05), ranging between 3.28 and 3.40% *w*/*w*. On the other hand, the grape juice yogurt presented significantly (*p* < 0.05) higher values of reducing sugars than the other yogurts throughout storage. In addition, after day 14 of storage, although CY, AY, and BY presented similar values of reducing sugars, GY presented significantly higher values due to the initial high sugar content of grape juice. 

It is well established that the excess consumption of free sugars increases the risk of obesity and dental caries and, therefore, the World Health Organization and several government agencies around the world have strongly recommended their reduction in foods [36]. In the present study CY, AY, and BY contained the usual content of sugars for set type yogurts and met the 5% *w*/*w* maximum required for a “low-sugar” claim under EU regulations [37]. GY contained higher content of sugars, especially at the first days of storage, but after the 14th day, it may also be characterized as “low-sugar” food.

Syneresis or whey separation is one of the most common defects in fermented milk products and especially set type yogurts. This defect may limit the shelf life of the product and affects its acceptability by the consumers due to the undesirable appearance. Usually, the consumers correlate syneresis with potential microbial infection of the yogurt [20,24,35]. Previous studies have correlated the addition of fruit juices with reduced viscosity and increased syneresis of yogurts [19,20]. More specifically, an increase in yogurt syneresis was observed with the increased content of carrot juice [20]. In order to reduce the extent of syneresis, several approaches may be applied such as increasing milk solids or using stabilizers (i.e., starch, gelatin, and vegetable gum) [24]. Gelatin is known to enhance the water holding capacity (WHC) of the gels and, therefore, it is usually used as stabilizer in yogurts. For example, the combined used of gelatin ≥ 1%, milk protein concentrate, and skim milk powder resulted in limited serum expulsion [38]. The same was observed when carrot juice was added (20% *v*/*v*) in yogurts and the addition of gelatin (0.7%) led to similar syneresis with the CY without juice [20]. However, the consumer demand for natural products makes the use of additives like gelatin unfavorable, while in some countries, it is forbidden [24]. 

In the present study, in order to overcome the abovementioned problems of syneresis and to avoid additives, the use of exopolysaccharide (EPS)-producing starter culture and the addition of skim milk powder were evaluated. The values of WHC and syneresis of yogurts during 28 days of storage are presented in Table 2. No significant (*p* > 0.05) differences were observed between the yogurts with different juices, presenting similar values with the control sample. In addition, the effect of storage was also insignificant in most cases, and a slight, but not significant, reduction of WHC and subsequent increase in syneresis were observed with storage time. These results prove that the combination of EPS-producing starter and skim milk powder was ideal to overcome the syneresis problems observed in other studies with fruit juices. Furthermore, the addition of juice at the same time as starter culture also seems to play a significant role in these results. The results of syneresis are significantly lower than those observed in other studies with yogurts fortified with carrot juice and gelatin [20] and concentrated grape juice [19].

Table 3 shows total phenolic content (TPC) and antioxidant activity (DPPH^●^ radical scavenging activity) of yogurts produced with the addition of different fruit juices. The use of different fruit juice significantly (*p* < 0.001) affected the TPC. More specifically, BY presented the highest content, while the lowest was detected in CY. The addition of grape or aronia juice had similar values but significantly higher than the CY. The fortification with fruit juices increased TPC from 30 to 33% in the case of grape and aronia juice and up to 49% in the case of blueberry. The high TPC of CY (43.3 μg GAE g^−1^) was expected since phenolic compounds are usually found in considerable amounts in ruminant milk (μg g^−1^) [39]. The majority of these are derived from the animal feed, although a proportion may be the products of amino acid catabolism. During storage, TPC remained stable in the case of CY, but a slight and not significant (*p* > 0.05) decrease was observed in the case of fruit juice-fortified yogurts (up to 5%). This observation is in accordance with previous studies on pomegranate juice [40,41] and grape and callus extracts [42] in yogurts, and it may be attributed to the formation of complexes between phenolic compounds and milk proteins that may affect phenolics recovery and activity [43]. Indeed, the phenolic group is an excellent hydrogen donor that forms hydrogen bonds with the carboxyl group of the protein and, therefore, several studies have demonstrated that phenolic compounds may interact with proteins in several ways, both reversibly and irreversibly [44].

The in vitro antioxidant activity of yogurts (day 1) was measured using the DPPH^●^ free radical scavenging activity and expressed as % reduction and as μmol Trolox equivalents 100 g^−1^ (Table 3). The same pattern was observed as in the case of TPC. CY presented the lowest antioxidant activity, while BY presented the highest followed by AY and GY. Similar results were observed in previous studies with aronia juice [28]. A decrease in antioxidant activity was observed with storage, which is in accordance with previous studies on pomegranate juice [41] and grape and callus extracts [42]. Oppositely, studies carried out in yogurts fortified with pomegranate juice [40] showed a tendency to increase antioxidant activity during storage. In general, the interactions of phenolic compounds and proteins are known to affect among others the content, antioxidant capacity, and bioavailability of phenolic compounds in foods [44] and thus explain the results of the present study.

### 3.3. Color Characteristics

Color is a very important characteristic of foods since it is the first sensory attribute that consumers observe for foods and may affect their preference. The results of color changes in yogurt added with fruit juices measured as L*, a*, b*, chroma, and hue angle values are shown in Table 4. The L* values, presenting the lightness of the product, showed that the addition of fruit juices decreased significantly (*p* < 0.05) the lightness of yogurts. The average values of L* during storage showed that CY was lighter (82.57 ± 2.51), followed by AY (77.00 ± 5.03) and GY (74.54 ± 2.77), while BY was the least light (71.52 ± 3.75). Storage did not affect the values of CY but significantly (*p* < 0.05) affected the fruit yogurts. More specifically, GY presented an increase in L* value and therefore became lighter while AY presented a decrease, becoming darker. 

The addition of fruit juices, and especially blueberry juice, resulted in an increased a* value (redness) of yogurts. More specifically, the a* value of yogurt was increased significantly (*p* < 0.05) from –2.70 in the CY to 1.21, 1.64, and 7.35 for AY, GY, and BY, respectively, indicating an increase in the red color. Storage did not affect the a* value of CY. However, a slight decrease in red color of GY and an increase in that of AY was reported, making the AY more red than GY at the end of storage time. The b* value (yellowness) of yogurt was decreased by the addition of fruit juices and especially in the case of blueberry. Similar results were reported in a previous study with aronia juice in yogurt. More specifically, the results showed that as the concentration of aronia juice increased, the yogurt became darker, redder, and less yellow compared to the control yogurt [28].

Regarding chroma, BY presented the highest values, followed by CY, AY and, finally, GY. Therefore, BY is characterized as the brightest while GY was the dullest. Storage time did not affect CY though GY and BY became brighter and AY duller with storage time.

Hue values are stepped counterclockwise on a 360° chromatic circle, where 0, 90, 180, and 270 represent the red-purple, yellow, bluish-green, and blue hues, respectively [45,46]. In the present study, the addition of fruit juices significantly affected (*p* < 0.001) the hue values of yogurts. More specifically, CY presented a white hue (115.0 ± 0.3°), AY a light red hue (76.7 ± 2.6°), GY a darker red (59.5 ± 0.9°), and BY a red/purple hue (7.4 ± 0.4°). Hue values of CY were similar (103–110°) with those of previous studies [30,47]. Storage at 4 °C, significantly affected hue values of all yogurts and this effect was more significant in fruit juices (*p* < 0.001) than for the CY (*p* = 0.02). An increase in hue values was observed in CY and GY while a reduction was observed in the case of AY. In general, after 28 days of storage, GY and AY presented similar values of hue. 

### 3.4. Starter Culture Viability

In the present study, the starter culture that was used contained increased numbers of *S. thermophilus* strains and low content of *Lactobacillus delbrueckii* ssp. *bulgaricus*. This was also confirmed during the microbiological analysis of the starter culture that was added in each yogurt formulation. The initial numbers were 7.50 ± 0.20 and 2.50 ± 0.20 log CFU g^−1^ for *S. thermophilus* and *L. bulgaricus*, respectively. Since the nature of the culture provides a low content of *L. bulgaricus*, the present study focused only on the trend of *S. thermophilus* viability during processing and storage. The numbers of *L. bulgaricus* decreased as was also reported in numerous studies with yogurts [35] and especially in samples with fruit juices [48].

**Figure 3:**
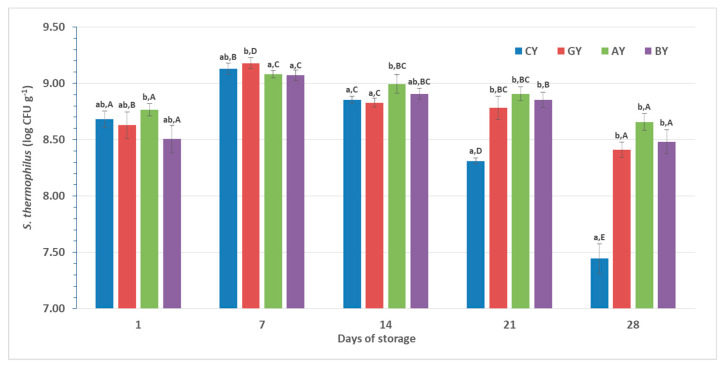
Viability of *S. thermophilus* in yogurts during refrigerated storage; CY = control yogurt; GY = yogurt with grape juice; BY = yogurt with blueberry juice; AY = yogurt with aronia juice; ^a,b^ Different lowercase letters in the columns, at the same day of storage, indicate significant differences (*p* < 0.05); ^A–E^ Different uppercase letters in the columns, at the same yogurt, indicate significant differences (*p* < 0.05).

In the dairy industry, it is very important to facilitate the survival of yogurt starter bacteria during the processing and storage of yogurts. Therefore, FAO/WHO has established a requirement of a minimum number of viable cells of yogurt starter bacteria of 10^7^ cells g^−1^ during consumption [49]. In the present study, these numbers were higher than 10^8^ cells g^−1^ after 28 days of storage for all yogurt formulations with juices and higher than 10^7^ cells g^−1^ for CY (Figure 3). 

*Streptococcus thermophilus* retained high viable counts during the storage period in yogurts with and without fruit juices. In general, this microorganism has been proven as capable of surviving in sufficient numbers even at low temperatures during yogurt production with milk of several origins [50,51]. A previous study [48] showed that the addition of fruit juices (usually with low pH) after yogurt formation produces a negative impact on starter culture bacteria, probably due to acid injury, in accordance with the findings of [52]. In order to avoid that, in the present study, fruit juices were added in milk prior to the addition of starter culture and the final pH after fermentation was the same in all cases, 4.67 ± 0.02.

The viability of *S. thermophilus* during storage presented the same trend in all cases with and without fruit juice. This trend is also confirmed in previous studies that reported an increase up to the first week of storage and then a continuous slight reduction [53,54]. More specifically, after the addition of starter culture the numbers of *S. thermophilus* increased up to 1 log CFU g^−1^ during the first day of storage, and with an additional 0.5 log CFU g^−1^ at the end of the first week (a total of 1.5 log CFU g^−1^). After the first week, a reduction of 0.5 log CFU g^−1^ until the end of 4 weeks was reported. 

After 28 days of storage, the numbers of *S. thermophilus* were significantly (*p* < 0.05) higher in the yogurts fortified with fruit juices compared to CY. This may be attributed to the increased content of phenolic compounds that may act as prebiotic and enhance the viability of *S. thermophilus*. Indeed, phenolics have been included in the recent definition of prebiotics [55] and recent studies have evaluated their prebiotic potential in in vitro studies [56,57].

The viability of *S. thermophilus* in yogurts during storage is considered very important since, in recent years, these strains have been demonstrated to have probiotic potential [58,59]. Even if most of the *S. thermophilus* strains appeared to be sensitive to acidic pH and bile salts, human studies have established their ability to survive human gastrointestinal transit and transiently colonize the human intestinal tract [60].

## 4. Conclusions

The present study clearly showed that the addition of fruit (aronia, blueberry, and grape) juices had no significant effect on yogurt physicochemical characteristics apart from color (increased red). On the other hand, they significantly increased total phenolic content and antioxidant activity. These results are considered very promising for the design and development of novel dairy products with increased functional characteristics. However, more studies are needed to evaluate possible alterations on organoleptic characteristics, flavor compounds, and possible interactions with probiotic cells. In addition, in vivo studies would also be helpful to clarify the potential health benefits of such products.

## Figures and Tables

**Table 1:** Effect of storage on pH values, acidity, and reducing sugars of yogurts.

Analyses	Days	CY	GY	AY	BY
pH	1	4.64 ± 0.03 ^c,A^	4.66 ± 0.01 ^d,A,B^	4.72 ± 0.01 ^c,B,C^	4.76 ± 0.01 ^c,C^
	7	4.57 ± 0.00 ^b,c,A^	4.59 ± 0.01 ^c,A^	4.51 ± 0.01 ^b,B^	4.51 ± 0.01 ^b,B^
	14	4.50 ± 0.00 ^a,b,A^	4.48 ± 0.01 ^b,A^	4.50 ± 0.01 ^b,A^	4.49 ± 0.00 ^b,A^
	21	4.45 ± 0.06 ^a,A^	4.39 ± 0.01 ^a,A^	4.40 ± 0.01 ^a,A^	4.38 ± 0.00 ^a,A^
	28	4.39 ± 0.01 ^a,A^	4.36 ± 0.01 ^a,A^	4.38 ± 0.01 ^a,A^	4.37 ± 0.01 ^a,A^
Acidity (% *w*/*w* lactic acid)	1	1.02 ± 0.00 ^a,B^	1.03 ± 0.01 ^a,b,B^	0.85 ± 0.01 ^a,A^	0.86 ± 0.01 ^a,A^
	7	1.03 ± 0.02 ^a,A,B^	0.99 ± 0.02 ^a,A^	1.06 ± 0.02 ^b,B^	1.08 ± 0.01 ^c,B^
	14	1.04 ± 0.02 ^a,A^	1.00 ± 0.01 ^a,A^	1.03 ± 0.01 ^b,A^	1.02 ± 0.01 ^b,A^
	21	0.99 ± 0.02 ^a,A^	1.00 ± 0.00 ^a,A^	1.01 ± 0.04 ^b,A^	1.00 ± 0.01 ^b,A^
	28	1.03 ± 0.01 ^a,A^	1.07 ± 0.01 ^b,A^	0.99 ± 0.04 ^b,A^	1.00 ± 0.00 ^b,A^
Reducing sugars (% *w*/*w* lactose)	1	5.00 ± 0.10 ^a,B^	6.18 ± 0.16 ^a,C^	4.22 ± 0.12 ^a,A^	4.35 ± 0.03 ^a,A^
	7	4.19 ± 0.39 ^b,A,B^	5.10 ± 0.07 ^b,B^	3.64 ± 0.10 ^a,b,A^	3.55 ± 0.11 ^b,A^
	14	3.49 ± 0.00 ^b,c,A^	4.39 ± 0.14 ^c,B^	3.48 ± 0.24 ^a,b,A^	3.66 ± 0.26 ^b,A^
	21	3.30 ± 0.08 ^c,A^	4.20 ± 0.02 ^c,d,B^	3.64 ± 0.14 ^b,A^	3.56 ± 0.00 ^b,A^
	28	3.39 ± 0.00 ^c,A^	3.98 ± 0.00 ^d,B^	3.40 ± 0.13 ^b,A^	3.28 ± 0.23 ^b,A^

^a–c^ Means within a column at the same analysis with different lowercase superscripts differ significantly (*p* < 0.05); ^A–C^ Means within a row with different uppercase superscripts differ significantly (*p* < 0.05); (CY = control yogurt; GY = yogurt with grape juice; BY = yogurt with blueberry juice; AY = yogurt with aronia juice).

**Table 2:** Effect of storage on water holding capacity (WHC) and syneresis of yogurts.

Analyses	Days	CY	GY	AY	BY
WHC (%)	1	69.6 ± 3.0 ^a^	72.2 ± 4.2 ^a^	67.6 ± 0.1 ^a,b^	66.6 ± 0.1 ^a,b^
	7	65.4 ± 0.1 ^a^	66.4 ± 3.5 ^a^	68.9 ± 3.3 ^b^	66.9 ± 1.1 ^b^
	14	65.9 ± 0.5 ^a^	67.7 ± 2.1 ^a^	68.9 ± 0.7 ^b^	69.1 ± 1.5 ^b^
	21	61.9 ± 3.6 ^a^	64.3 ± 1.4 ^a^	64.6 ± 0.4 ^a^	66.2 ± 2.3 ^a,b^
	28	62.0 ± 0.1 ^a^	62.5 ± 0.1 ^a^	61.9 ± 1.7 ^a^	61.2 ± 1.1 ^a^
Syneresis (%)	1	30.0 ± 0.0 ^a^	31.0 ± 1.4 ^a^	38.0 ± 2.1 ^a^	38.3 ± 4.6 ^a^
	7	35.5 ± 0.7 ^a^	38.5 ± 0.7 ^a^	37.5 ± 2.1 ^a^	37.5 ± 2.1 ^c^
	14	33.8 ± 3.2 ^a^	33.0 ± 3.5 ^a^	33.0 ± 1.4 ^a^	37.0 ± 4.2 ^b^
	21	38.5 ± 3.5 ^a^	31.4 ± 2.8 ^a^	37.0 ± 4.2 ^a^	34.0 ± 2.8 ^b^
	28	34.6 ± 0.9 ^a^	33.8 ± 3.9 ^a^	39.0 ± 1.4 ^a^	40.5 ± 3.5 ^b^

^a,b^ Means within a column at the same analysis with different superscripts differ significantly (*p* < 0.05); (CY = control yogurt; GY = yogurt with grape juice; BY = yogurt with blueberry juice; AY = yogurt with aronia juice).

**Table 3:** Antioxidant activity and total phenolic content of yogurts.

Yogurt	DPPH^●^		Total Phenolic Content μg GAE g^−1^
	%	μmol TE 100 g^−1^	
CY	17.5 ± 1.2 ^a^	14.7 ± 0.9 ^a^	43.3 ± 0.2 ^a^
GY	27.1 ± 2.6 ^b^	21.5 ± 1.8 ^b^	57.6 ± 0.3 ^b^
AY	39.2 ± 1.1 ^c^	30.0 ± 0.8 ^c^	56.5 ± 1.1 ^b^
BY	43.5 ± 0.2 ^d^	33.0 ± 0.2 ^d^	64.6 ± 0.6 ^c^

^a–d^ Means within a column at the same analysis with different superscripts differ significantly (*p* < 0.05); (CY = control yogurt; GY = yogurt with grape juice; BY = yogurt with blueberry juice; AY = yogurt with aronia juice; TE = Trolox equivalents; GAE = gallic acid equivalents).

**Table 4:** Effect of storage on color characteristics of yogurts.

Analyses	Days	CY	GY	AY	BY
L*	1	81.43 ± 7.13 ^a^	72.01 ± 3.23 ^a^	81.59 ± 1.87 ^b^	70.94 ± 2.69 ^a^
	7	81.46 ± 7.22 ^a^	71.99 ± 3.30 ^a^	83.32 ± 7.22 ^b^	76.76 ± 2.39 ^c^
	14	81.83 ± 2.49 ^a^	73.79 ± 5.67 ^a^	73.00 ± 2.58 ^a^	66.21 ± 3.18 ^b^
	21	81.11 ± 6.34 ^a^	77.85 ± 3.83 ^a^	73.85 ± 0.26 ^a^	71.69 ± 0.82 ^a^
	28	87.04 ± 5.48 ^a^	77.07 ± 0.03 ^a^	73.22 ± 0.22 ^a^	71.98 ± 0.15 ^a^
a*	1	−2.78 ± 0.26 ^a^	1.64 ± 0.05 ^a^	1.21 ± 0.25 ^a^	7.35 ± 0.17 ^a^
	7	−2.71 ± 0.37 ^a^	1.62 ± 0.10 ^a^	1.89 ± 0.30 ^b,c^	8.74 ± 0.27 ^c^
	14	−2.64 ± 0.16 ^a^	1.48 ± 0.16 ^a^	1.73 ± 0.15 ^b^	7.61 ± 0.51 ^a,b^
	21	−2.72 ± 0.31 ^a^	1.48 ± 0.06 ^a^	1.98 ± 0.03 ^b,c^	8.02 ± 0.11 ^b^
	28	−2.71 ± 0.22 ^a^	1.51 ± 0.07 ^a^	2.09 ± 0.03 ^c^	7.94 ± 0.06 ^b^
b*	1	5.95 ± 0.49 ^a^	2.78 ± 0.01 ^a^	5.09 ± 0.04 ^c^	0.96 ± 0.03 ^a^
	7	5.81 ± 0.77 ^a^	2.82 ± 0.01 ^a,b^	4.72 ± 0.45 ^b,c^	1.13 ± 0.05 ^a,b^
	14	3.59 ± 0.22 ^a^	2.91 ± 0.34 ^a,b,c^	4.17 ± 0.26 ^a^	0.78 ± 0.05 ^a^
	21	5.72 ± 0.76 ^a^	3.20 ± 0.20 ^c^	4.27 ± 0.26 ^a,b^	1.32 ± 0.32 ^b^
	28	5.60 ± 0.26 ^a^	3.12 ± 0.04 ^b,c^	4.30 ± 0.19 ^a,b^	1.40 ± 0.33 ^b^
C*	1	6.57 ± 0.55 ^a^	3.23 ± 0.02 ^a^	5.24 ± 0.09 ^c^	7.41 ± 0.02 ^a^
	7	6.41 ± 0.85 ^a^	3.25 ± 0.04 ^a^	5.08 ± 0.53 ^b,c^	8.82 ± 0.26 ^d^
	14	6.19 ± 0.26 ^a^	3.26 ± 0.37 ^a^	4.51 ± 0.30 ^a^	7.65 ± 0.51 ^a,b^
	21	6.33 ± 0.82 ^a^	3.53 ± 0.20 ^a^	4.71 ± 0.22 ^a,b^	8.13 ± 0.16 ^c^
	28	6.22 ± 0.33 ^a^	3.47 ± 0.07 ^a^	4.78 ± 0.16 ^a,b,c^	8.06 ± 0.10 ^b,c^
°hue	1	115.0 ± 0.3 ^a^	59.5 ± 0.9 ^a^	76.7 ± 2.6 ^d^	7.4 ± 0.4 ^a,b^
	7	115.0 ± 0.1 ^a^	60.2 ± 1.6 ^a^	68.3 ± 1.3 ^c^	7.3 ± 0.6 ^a,b^
	14	115.3 ± 0.5 ^a^	63.0 ± 0.5 ^b^	67.5 ± 0.5 ^b,c^	5.9 ± 0.8 ^a^
	21	115.5 ± 0.5 ^a,b^	65.2 ± 0.7 ^c^	65.1 ± 1.7 ^a,b^	9.3 ± 2.1 ^b,c^
	28	115.8 ± 0.8 ^b^	64.2 ± 0.8 ^b,c^	64.0 ± 1.1 ^a^	10.0 ± 2.3 ^c^

^a–c^ Means within a column at the same analysis with different superscripts differ significantly (*p* < 0.05).
